# Supplementary material for: Thymic Exhaustion and Increased Immune Activation Are the Main Mechanisms Involved in Impaired Immunological Recovery of HIV-Positive Patients under ART
Source: Viruses. 2023 Feb 5;15(2):440. doi: 10.3390/v15020440 (PMC9961132; doi:10.3390/v15020440)
Supplement: Supplementary file 1 [file viruses-15-00440-s001.zip › Supplementary Figure S1 - GatingStrategy.pdf]

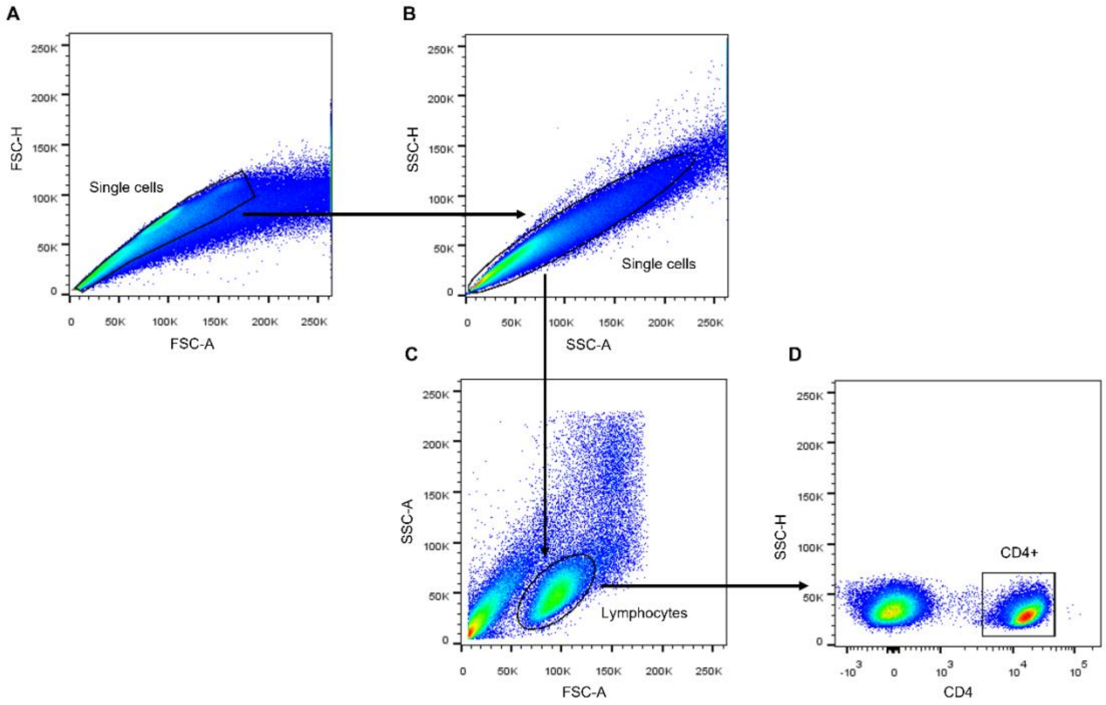

Supplementary Figure S1. Representative flow cytometry plots illustrating the gating strategy for T-lymphocytes. Two initial gating were performed to identify single cells by forward scatter height and area (FSC-H and FSC-A, **(A)**) and side scatter height and area (SSC-H and SSC-A, **(B)**). Lymphocytes were then selected from singles cells based on SSC-A and FSC-A **(C)**. Cells expressing CD4+ were selected to further analyses **(D)**.
