# Supplementary material for: Thymic Exhaustion and Increased Immune Activation Are the Main Mechanisms Involved in Impaired Immunological Recovery of HIV-Positive Patients under ART
Source: Viruses. 2023 Feb 5;15(2):440. doi: 10.3390/v15020440 (PMC9961132; doi:10.3390/v15020440)
Supplement: Supplementary file 1 [file viruses-15-00440-s001.zip › Supplementary Table S1.pdf]

Coinfections

The ART-treated HIV-positive patients enrolled in our study presented no active coinfection during ART, regarding hepatitis B and C virus (HBV and HCV), cytomegalovirus (CMV), toxoplasmosis, human T-cell lymphotropic virus types I and II (HTLV-I/II), and herpes simplex virus types 1 and 2 (HSV-1/2) based on clinical diagnosis for Immunoglobulin M tests. For HBV infection, 7.7% and 3.2% were immune by natural past infection whereas 7.7% and 35.5% were immune by vaccination in INR and IR groups, respectively, and 23.1% (INR) and 6.4% (IR) were susceptible – did not present evidence for contact with HBV. Evidence for syphilis infection was present in 23.1% (n=3) of patients in the INR group and 16.1% (n=5) in the IR group, as detected by VDRL test. In relation HSV 1/2, CMV and toxoplasmosis infections, there was presence of past coinfections revealed by immunoglobulin G tests (Supplementary Table S1). However, the coinfections had no influence over immunological response in this study (syphilis: P=0.706; CMV: P=0.706 and toxoplasmosis: P=0.214).

Supplementary Table S1. Coinfections serology status of HIV-positive patients during ART.

Coinfections Serology Status	INR n=13 (%)	IR n=31 (%)	*P* *
Syphilis	3 (23.1)	5 (16.1)	0.676
HSV 1/2[a]	2 (15.4)	8 (25.9)	0.697
Toxoplasmosis[a]	4 (30.8)	10 (32.2)	1.000
CMV[b]	8 (61.5)	19 (61.3)	1.000

* Fisher exact test.

[a] Immunoglobulin G tests.

CMV: cytomegalovirus; HSV 1/2: herpes simplex virus types 1 and 2; INR: immunological non-responders; IR: immunological responders.
